# Utilisation of health services fails to meet the needs of pregnancy-related illnesses in rural southern Ethiopia: A prospective cohort study

**DOI:** 10.1371/journal.pone.0215195

**Published:** 2019-12-04

**Authors:** Moges Tadesse Borde, Eskindir Loha, Kjell Arne Johansson, Bernt Lindtjorn

**Affiliations:** 1 School of Public and Environmental Health, College of Medicine and Health Sciences, Hawassa University, Hawassa, Ethiopia; 2 Centre for International Health, University of Bergen, Bergen, Norway; 3 School of Public Health, College of Medicine and Health Sciences, Dilla, Dilla University, Ethiopia; 4 Department of Infectious Disease Epidemiology, London School of Hygiene and Tropical Medicine, London, England, United Kingdom; 5 Department of Global Public Health and Primary Care, University of Bergen, Bergen, Norway; African Population and Health Research Center, KENYA

## Abstract

Although maternal survival has improved in the last decades, evidence on illnesses and the use of health services during pregnancy remains scarce. Therefore, we aimed to assess the incidence and risk factors for illnesses among pregnant women and measure the use of health services. A prospective cohort study was conducted in three kebeles in rural southern Ethiopia among 794 pregnant women from May 2017 to July 2018. Each woman was followed every two weeks at home. Poisson and survival regression models were used for analysis. The incidence rate of episodes of illnesses was 93 per 100 pregnant-woman-weeks (95%CI: 90.6, 94.2), with an average of eight episodes of illnesses per woman. Anaemia accounted for 22% (177 of 794 women), and hypertension 3% (21 women of 794 women). However, utilization of health services for any illness episodes was only 8% (95%CI: 7.6%, 8.9%). The main reasons for not using health services were that the women thought the illness would heal by itself, women thought the illness was not serious, women could not afford to visit the health institutions, or women lacked confidence in the health institutions. The risk factors for illnesses are having many previous pregnancies in life time (ARR = 1.42; 95%CI = 1.02, 1.96), having history of stillbirth (ARR = 1.30; 95%CI = 1.03, 1.64), having history of abortion (AHR = 1.06; 95%CI = 1.02, 1.11), and walking more than 60 minutes to access the nearest hospital (AHR = 1.08; 95%CI = 1.03, 1.14). The risk factors for low use of health services are also having history of abortion (AHR = 2.50; 95%CI = 1.00, 6.01) and walking more than 60 minutes to access the nearest hospital (AHR = 1.91; 95%CI = 1.00, 3.63). Rural Ethiopian pregnant women experience a high burden of illness during pregnancy. Unfortunately, very few of these women utilize health services.

## Background

In 2016, the United Nations reported that maternal survival has improved in the last decades [1]. However, there is scarce evidence on the occurrence of illness among pregnant women and their subsequent use of health services. In fact, one woman dies out of 20 women who suffer from illnesses during pregnancy. Thus, it is critical to obtain more precise estimates of illness burden during pregnancy from low resource settings [2].

Although ensuring healthy lives and promoting the well-being of women constitutes one of the major agenda items in Sustainable Development Goal Number 3 [3], the illness burden in developing countries among pregnant women remains high [4]. Globally, 10 to 20 million women suffer from pregnancy and childbirth-related illnesses annually [5]. Approximately 25% of pregnant women in developing countries [6] and 14% in Ethiopia in 2013 reported at least one type of illness during pregnancy [7].

Appropriate and timely healthcare-seeking and use of health services are essential for healthy living. The utilization of health services for illnesses during pregnancy is dependent on the women’s socioeconomic and demographic environment [8], such as the woman’s age, educational status, occupation, access, and travel time to a health facility [9]. Illnesses among pregnant women are associated with both increased maternal deaths and stillbirths [10,11]. In 2017, 56 million pregnant women experienced spontaneous abortion globally, and 49 million of these were in low-income countries [12]. In Ethiopia, in 2017, the rate of spontaneous abortion was 28 per 1000 women [13]. In 2015, the global number of stillbirths was estimated to be 2.6 million [13], and Ethiopia accounted for 4% of these (97,000 stillbirths) [13]. In addition, approximately 42% of pregnant women in developing countries were estimated to suffer from anaemia in 2017 [14], and 25% of Ethiopian pregnant women had anaemia [15]. Although iron-folic-acid supplementation is recommended for prevention or treatment of anaemia to all pregnant women who give birth [16], limited data exist concerning the uptake of iron-folic-acid supplementation during the antenatal and postpartum period from Ethiopia [15]. Hypertension is also common during pregnancy and occurs among 10% of pregnant women globally [17] and 6% in Ethiopia [18].

The causes and burdens of illnesses during pregnancy and the use of available health services in Ethiopia have not yet been well described. Therefore, we aimed to assess the incidence and risk factors for illnesses among pregnant women in rural communities in southern Ethiopia and measure the use of health services.

## Materials and methods

### Study design

A prospective cohort study was carried out among 794 pregnant women attending antenatal care (ANC). In 2016, the proportion of pregnant women attending at least one antenatal care in Ethiopia was 62% [19]. The healthcare system in Ethiopia is organized based on the type of care provided. For example, primary healthcare comprises one primary care unit (health centre) with five health posts, secondary healthcare includes zonal and regional hospitals, and tertiary care includes a central and teaching hospital. Primary healthcare utilizes the health extension package as an approach that focuses on communicable diseases, maternal health, common nutritional disorders, hygiene, and environmental health. Maternal and child healthcare, immunization against childhood illnesses, and family planning and reproductive health, infectious diseases, including tuberculosis, malaria and control of sexually transmitted infections and HIV/AIDS, are also critical areas to address. Pregnant women primarily use health extension workers at health posts. If the case is serious, the health post may refer them to a health centre.

According to the national antenatal care guidelines in Ethiopia, a focused ANC visit is advised to take place at least four times during a pregnancy. The main components of antenatal care include protection at birth from tetanus, blood pressure measurement, nutritional counselling, iron-folate supplementation, and information about the danger signs of pregnancy complications [19].

In 2013, more than 80% of the population lived in rural Ethiopia, 26% of the rural residents lived on less than $1 per day, and 77% of rural women needed to travel more than 20 km to get to a hospital [20].

### Setting

The Wonago district (zone) is located 420 km far away from the capital, Addis Ababa. It has 15 rural and four urban kebeles. A kebele is part of a district (wereda), which is the lowest administrative unit in Ethiopia and comprises approximately 1000–1500 households (an average of 5000–7500 people) [21]. In 2017, the total population of the district was estimated to be 145,000 people [22]. The major ethnic group was the Gedeo people, and the population density was 980 persons per km^2^. Agriculture is the dominant means of livelihood. The district has six health centres, 20 health posts, and two private clinics.

Three kebeles (i.e., Hase-Haro, Mekonisa, and Tumata-Chiricha) were randomly selected by the lottery method from Wonago district, which is located in the Gedeo zone in southern Ethiopia. The recruitment of pregnant women started in May 2017 and follow-up ended in July 2018. The three kebeles were similar in socio-demographic and economic features to most rural areas in Ethiopia. Mekonisa kebele has one health centre and two health posts, Tumata-Chiricha kebele has one health post, and Hase-Haro kebele has one health centre and one health post. In 2017, the estimated total population of the three kebeles was 29,780 people [22], and the crude birth rate of rural people in Ethiopia was 33.2 per 1000 population [19]. The proportion of observed (number of pregnant women included in our study) to expected (total number of pregnant women) antenatal care visits was 80.3% from the three kebeles, and varied from 70.6% to 85.2%. There is no significant difference between the expected and actual births from the three kebeles, x^2^ = 4.8618(2), p-value = 0.09 (Table 1).

**Table 1:** Expected and actual pregnancies from each kebele.

Kebele	Total Population	Expected births*	Actual births sampled for the study	Births included in the final analysis	% of births included in the study to expected births	X^2^ test and p-value
Hase-Haro	9733	323	251	228	70.6	X^2 =^ 4.8618, *p*-value = .09
Mokonissa	13746	456	441	388	85.1	
Tumata Chiricha	6301	209	204	178	85.2	
Total	29780	989	896	794	80.3	

*The number of expected pregnancy calculated based on EDHS 2016 [19]

### Participants

Pregnant women attending ANC at health posts formed the study population. All women in the reproductive age group in the selected kebeles were the source population. The pregnant women were recruited based on ANC visits mostly in the second trimester, which is estimated to be within 24–28 weeks of gestation according to EDHS 2016 [19]. All women were followed at regular intervals (every two weeks) at home based on a scheduled visit. The inclusion criteria were a pregnant woman who had attended two or more antenatal care visits to a health post. Exclusion criteria were a woman not living in the study area, or not found at home at the scheduled visit. Unfortunately, we did not include women with less than two antenatal visits, and this may have caused a selection bias, as we have discussed.

### Variables

#### Outcome variables

The criteria for identifying illnesses among pregnat women were decided prior to initiating the study. The concepts of illness have been previously used to indicate personal ailments (subjective undesirable state of health) [23]. A pregnant woman with an illness was identified using the illness category and/or by the recording of an associated disability (S1 Table). Illness identification criteria were based on general symptoms and screening of anaemia and hypertension. The symptoms and subsequent use of health services were recorded. Our assumption was that pregnant women would seek healthcare primarily due to medical problems. The primary outcome variable was the incidence of illnesses among pregnant women and measured among all participants. Subsequent use of health services was measured among those who had an illness during pregnancy.

#### Exposure variables

Pregnant women were followed over time to assess the occurrence of illnesses among pregnant women and subsequent use of health services. Women’s basic characteristics were defined as women’s age, women’s age at first marriage and at first birth (increase vs. decrease), marital status (ever married vs. not married), educational status (had formal education vs. had no formal education), occupation (others (daily labourer, farming, etc.) vs. domestic service), wealth index (rich vs. poor), total monthly household expenditure ($30+ vs. <$30), gravidity (multigravida vs. primigravida), parity (multipara vs. nullipara) prior viable pregnancy, birth interval (2+ years vs. <2 years), and history of abortion (yes vs. no) and stillbirth (yes vs. no). Community or kebele level exposure status of the pregnant women was defined as the type of road to the nearest health facility (asphalt vs. others), and walking distance to the nearest health post (30+ minutes vs. <30 minutes).

### Measurements

Questionnaires were used to assess illnesses amnog pregnat women and the use of health services. Blood pressure was measured by the Riester ri-champion^®^N digital apparatus (www.riester.de), and haemoglobin (Hgb) was measured by the HemoCue analyzer®Hb 301 System (www.hemocue.com).

Hgb values for pregnant women were categorized into no anaemia (> = 11 g/dl), mild anaemia (10–10.9 g/dl), moderate anaemia (7–9.9 g/dl), severe anaemia (4–6.9 g/dl), and very severe anaemia (< = 3.9 g/dl). As the concentration of Hgb declines during the first trimester, reaches its lowest point in the second trimester, and begins to rise again in the third trimester, due to physiological changes, Hgb values for pregnant women were determined at around the third trimester, or 27 gestational weeks or later [24].

Hypertension during pregnancy was classified as either a systolic blood pressure greater than 140 mmHg, or diastolic blood pressure greater than 90 mmHg, or both [25]. At the time of each visit, blood pressure (BP) readings were taken in at least one-minute intervals [26] between two consecutive readings, and their mean was recorded.

### Sample size

**Fig 1:**
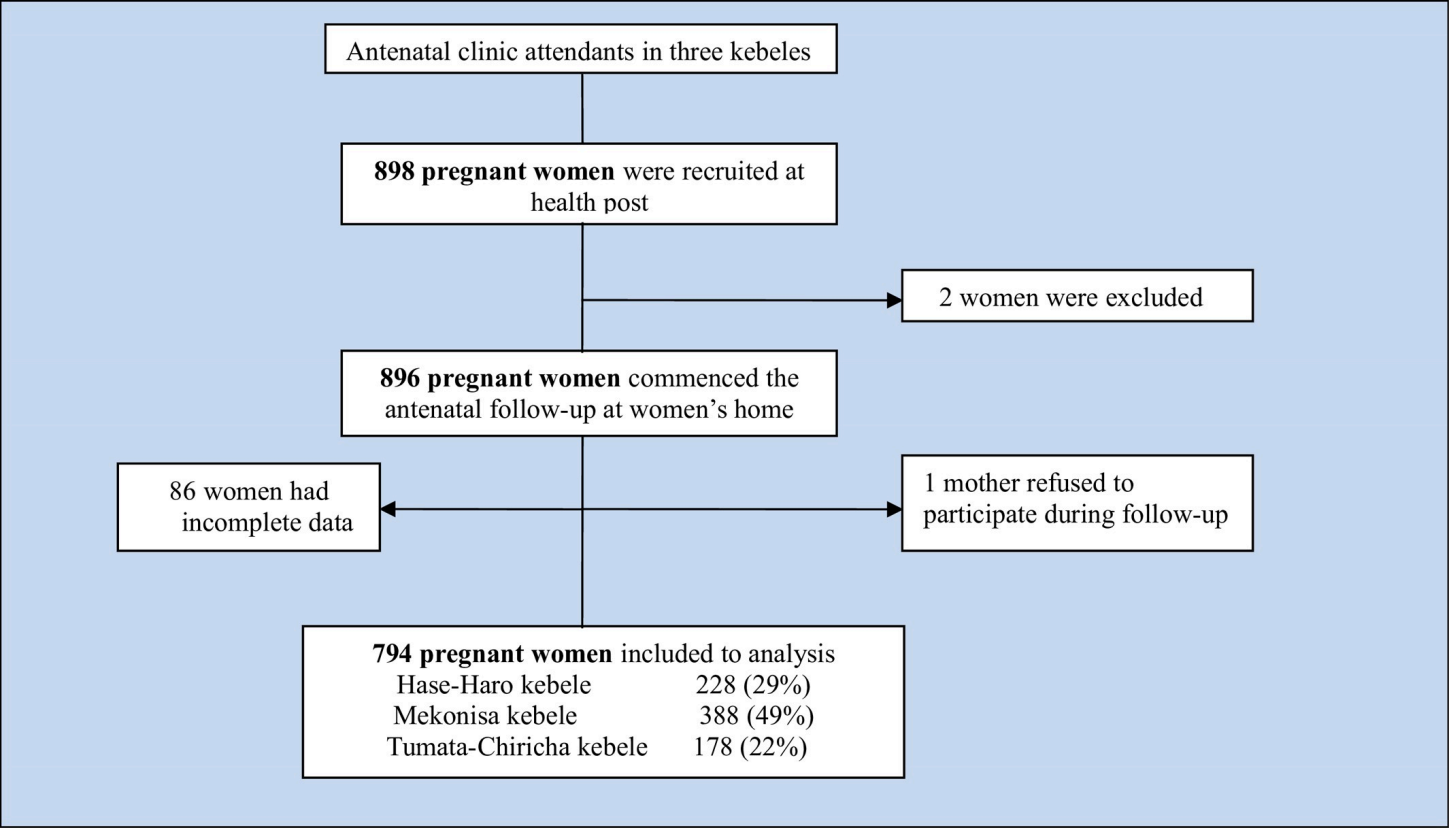
Flowchart of recruitment of pregnant women in rural southern Ethiopia, 2017/18.

The sample size was determined by Openepi software Version 3.03 (www.openepi.com). To obtain the maximum sample size, we used different socio-demographic factors as exposure variables and the incidence of illnesses among pregnant women as outcome variables. The following assumptions were made to assess the sample size: 15.5% of the incidence of illnesses among pregnant women, and 1.65 relative risk [7] among poor compared with rich women, with 95% confidence level, 80% power, and 1:1 ratio of unexposed to exposed. The sample size was estimated to be 898 after adding 10% for non-response (Fig 1).

### Quantitative variables

Continuous variables were assessed for symmetry, and parametric tests were used for normally distributed variables. For example, women’s age was categorised into the following groups (15–19, 20–24, 25–29, 30–34, and 35+ years), women’s age at first marriage (10–14, 15–19, 20–24, and 25–29), and women’s age at first birth (15–19, 20–24, and 25–29). Walking distance to the nearest health post was classified based on the mean of the sample in minutes (<30 and 30+), and household total daily expenditure categorized based on the $1 a day poverty line for developing countries [27] (<$30 and $30+ a month).

### Data collection

At baseline and during the follow-up period, we collected information on variables that are important for the study. We then assessed illness among pregnant women. The illnesses during pregnancy may occur once or multiple times. The follow-up time ranged from four weeks to 14 weeks. The data were collected at home every two weeks, and only one woman per household was included in the study. At each visit, data on the use of health services and reasons why they did not seek healthcare were collected from the women.

Data were collected using a validated interviewer-administered questionnaire, which was adapted from the “WHO maternal morbidity measurement tool pilot: study protocol” [2] by piloting the questionnaire. The pregnancy questionnaires were prepared in English, translated into the local languages, i.e., Gedeo language (S1 Questionnaire.rar) and Amharic (S2 Questionnaire.rar), and then back-translated into English (S3 Questionnaire.rar). A pre-test was conducted in a neighbouring kebele. The data collectors read the symptoms, and women indicated whether they had any of the symptoms before the current visit (in the past two weeks). Pregnant women were also asked to report any other health-related problems that they had experienced. The six data collectors were women, residents of the selected kebeles, and had completed grade 10.

### Operational definitions

Stillbirth: a baby born with no signs of life at or after 28 weeks' gestation [28]. Abortion: is the natural death of an embryo or fetus before it is able to survive independently before 28 weeks of gestation. Gravidity: all number of pregnancies in a lifetime (includes complete or incomplete). Parity: number of children previously borne by a woman (excludes abortions, but it includes stillbirths). Utilisation of health services: defined as the number of healthcare services used by persons for the purpose of curing illnesses. Recurrent events: an event (i.e., illness and use of health services) experienced repeatedly by pregnant women. These events could all be of the same type or different types. Repeated measures: a research design that involves multiple measures of the same variable taken on the same subjects either under different conditions or over two or more time periods. Therefore, in this study, we measured repeatedly the type of illnesses and use of health services during pregnancy. Multiple responses: refers to the situation in which people are allowed to tick or respond with more than one answer option for a question.

### Statistical methods

The data were entered in EpiData version 3.1 software (EpiData Association Odense, Denmark). Principal components analysis (PCA) was utilized to construct a wealth index of households based on 35 household assets and facilities. For this study we used two categories of quintiles (i.e. from 1st to 3rd quintiles categorized as poor, and the 4^th^ and 5^th^ quintiles categorized as rich). Descriptive statistical analysis was used to determine the distribution of the incidence of illnesses and the use of health services.

In this recurrent events analysis, the pregnant woman was at risk for the same or different events throughout the follow-up period, regardless of whether or not an event has occurred. Different pregnant women, of course, could have different numbers of events; some women had no illness or did not use health services, whereas others had many or did use health services. However, these different numbers of events that were observed across different pregnant women tended to follow a certain pattern that can be described using a Poisson distribution [29]. The recurrent event was described by estimating the mean cumulative function, which was the average number of cumulative events experienced by a pregnant woman in the study since the start of follow-up. The outcome (an event) was analysed as a count variable, and illnesses as exposure for use of health services were also analysed.

In this paper, two classes of statistical models were used to analyze recurrent event data: Poisson regression model and longitudinal techniques, which are an extension of the Cox-proportional hazards regression survival model. Cumulative number of events (counts) and event rates (total number of events divided by total follow-up period) by the end of the study were assessed using these two models.

Poisson regression is a technique that models the number of events (i.e., illnesses and use of health services) and the length of follow-up time. In the Poisson analysis method, all events were assumed to be independent. The Poisson model does not utilize all available data, but rather uses only one summary observation for each pregnant woman. The Poisson analysis method was done for the count data, including an offset for the time in the study, and event rates. Poisson logistic regression analysis was performed to analyse the difference in the proportion of pregnant women with illnesses at the end of the study. The data structure for fitting the Poisson regression model was composed of a column labelled “number of events” which was used as an outcome, and a column labelled “the length of follow-up time” which was used as an offset term. To account for the different lengths of follow-up between pregnant women, we included an offset term denoting the length of follow-up. In the dataset, each pregnant woman contributed one record.

In longitudinal techniques, the model does not use one observation for each pregnant woman, but instead all observations for each pregnant woman are used in the analysis. In such a long data structure, there was more than one record present for each pregnant woman. The time at risk is defined as the total time approach, in which the starting point for each period is the beginning of the study. The Prentice-Williams-Peterson (PWP) total time model is an extension of the Cox-proportional hazards regression survival model, which was utilized to analyze the repeated occurrence of events (recurrent events) over time, as there is a dependency of observations within a pregnant woman [30]. The PWP total time model considers each sequential event (first, second, etc.) separately because the time scale used in this model is the time from study entry. The model was stratified by event sequence, so that the baseline hazard function can differ between the sequential events. The PWP total time model allows any covariate to have different associations with different sequential events. The data structure for fitting the PWP total time model was composed of a column labelled “sequence number” which represents the order of time intervals for each pregnant woman which is unique to each sequential event and can be used to define the strata, the columns labelled “start-time” and “end-time” represents the start and end time of each interval, respectively, and the column “event indicator” represents whether an event occurs at the end of the time interval.

A multivariable regression model was carried out to identify independent predictors of the incidence of illnesses. The interaction effect of 20 exposure variables was examined, and no significant interaction effects were observed. The Hosmer and Lemeshow recommendations were used in the selection of the factors, which were P-values ≤0.2 in univariate analysis for multivariate analysis [31]. P-values ≤0.05 were used as cut-off points to determine statistical significance. Poisson regression and the PWP total time model survival model was fitted in STATA software version 15 (Stata Corp., LLC. College Station, Texas U.S.A.).

### Ethical considerations

This study was approved by the Institutional Ethical Review Board at Hawassa University, College of Medicine and Health Sciences (IRB/100/08), and by the Regional Committees for Medical and Health Research Ethics (REC) of western Norway (2016/1626/REK vest). Written permission letters remain from the Gedeo Zone Health Department and the Wonago Wereda (district) health office. Written informed consent was obtained from each mother after she had received an explanation of the purpose of the study. The privacy, anonymity, and confidentiality of study participants were maintained. If a woman was found to have an illness during pregnancy, the data collectors linked the patient with health extension workers in the kebele.

## Results

The response rate was 88.6% (794 of 896 women), and 11.4% (102 of 896 women) had incomplete data (86 women had defaulted, one died, one refused to participate, and 14 abortions occurred after week 21 and before 28 weeks of gestation) (Fig 1).

### Background characteristics of the pregnant women

Table 2 presents the background characteristics of the pregnant women. Almost all (99.6%; 791 of 794 women) were married, 63.7% (506 of 794 women) were illiterate, and 61.2% (486 of 794 women) were poor. The mean age was 25.3 years (range: 16, 45). The median walking distance to the nearest health post was 30 minutes (interquartile range, IQR: 20, 50). The median monthly household total expenditure was 42.4 USD (IQR: 30.3, 60.1), of which 87% was spent on food.

**Table 2:** Characteristics of the antenatal care study population in Wonago district, rural southern Ethiopia, May 2017 to July 2018 (= 794).

Socioeconomic characteristics		Frequency	Percent
Kebele/residence	Mekonisa	388	48.9
	Hase-Haro	228	28.7
	Tumata-Chiricha	178	22.4
Age of mother in years	15–19	106	13.4
	20–24	226	28.5
	25–29	289	36.4
	30–34	131	16.5
	35+	42	5.3
Marital status	Not married	3	0.4
	Ever married	791	99.6
Maternal educational status	Had no formal education	506	63.7
	Had formal education	288	36.3
Maternal occupation	Domestic service	734	92.4
	Others (daily labourer, farming, etc.)	60	7.6
Wealth index	Poor	333	41.9
	Rich	461	58.1
Type of road to the nearest health facility	Others	586	73.8
	Asphalt	208	26.2
Walking distance to the nearest health post in minutes	<30	437	55.0
	30+	357	45.0
Walking distance to the nearest health centre in minutes	<40	522	65.7
	40+	272	34.3
Walking distance to the nearest hospital in minutes	<60	335	42.2
	60+	459	57.8
Total household total expenditure per month	<$30	197	24.8
	$30+	597	75.2
**Demographic characteristics**		
Age of mother at first marriage in years	10–14	1	0.1
	15–19	714	89.9
	20–24	78	9.8
	25–29	1	0.1
Age of mother at first birth in years	15–19	451	56.8
	20–24	337	42.4
	25–29	6	0.8
Gravidity (no. of pregnancy)	Primigravida	185	23.3
	Multigravida	609	76.7
Parity (no. of birth)	Nullipara	187	23.6
	Multipara	607	76.4
Birth interval in years	<2	387	48.7
	2+	407	51.3
History of abortion	No	746	94.0
	Yes	48	6.0
History of stillbirth	No	756	95.2
	Yes	38	4.8

1 USD = 27.64 ETB on August 31, 2018.

The mean age of pregnant women at their first marriage was 18.1 years (range: 14, 28), and the mean age at first birth was 19.4 years (range: 16, 28). Approximately one-quarter (23.3%; 185 of 794 women) reported that the current pregnancy was their first pregnancy, and 54.3% (431 of 794 women) had two or more children.

### Birth outcomes

Approximately two-thirds (64.1%; 509 of 794 women) delivered at home, and of these, 96.3% (490 of 509 women) were attended by family members. There were 781 singleton and 13 multiple births. Vaginal deliveries accounted for 98.6% (783 of 794 women) of the deliveries. Fourteen women experienced abortions before 28 weeks of gestation (17.6 per 1000 pregnant women), and 26 deliveries resulted in a stillbirth (stillbirth rate 33.2 per 1000 births). We registered one maternal death. None of the women who experienced abortion used health services.

### Incidence of illnesses among pregnant women

Table 3 shows illnesses among pregnant women, the use of health services, and reasons for not using health services. Over an average follow-up of 9.9 weeks, there was a total of 6,705 illness episodes (minimum = 1, maximum = 45, and median = 6 episodes per pregnant woman). The total length of follow-up time for each of the types of illnesses was 7852 pregnant women-weeks. The incidence rate of episodes of illnesses was 92.6 per 100 pregnant-woman-weeks (95%CI: 90.6%, 94.2%) (5145 failures in multiple-failure-per-subject data over 5558 total analysis time at risk and under observation). Approximately 735 of 794 pregnant women experienced at least one type of illness during pregnancy with an average of eight episodes of illnesses per woman. During pregnancy, tiredness (72.4%), heartburn (62.5%), pain in the pelvic area (52.6%), severe headache (46.5%), and dizziness (42.9%) constituted the most common problems facing the women during pregnancy (Table 3).

**Table 3:** Illnesses among pregnant women, use of health services, and reasons for not using health services in rural southern Ethiopia, May 2017 to July 2018.

Types of illnesses	No. of women with illness	%	No. of women without illness	No. of events for illnesses (episodes of illness)	Length of follow-up time	No. of events for the use of health services (episodes of the use of health services)		Episodes of illness per woman	Reason for not using the health services at least once								
									Waited for self-recovery		The illness was not serious		Lack of money		Lack of trust		Total
						Yes	%	N(95%CI)*	Yes	%	Yes	%	Yes	%	Yes	%	
Tiredness	575	72.4	219	1277	7852	100	7.8	2.2(1.8,2.6)	253	13.4	214	14.6	93	10.9	13	9.4	573
Heartburn	496	62.5	298	1097	7852	59	5.4	2.2 (1.8,2.6)	205	10.9	208	14.2	76	8.9	12	8.7	501
Pain in the pelvic area	418	52.6	376	742	7852	62	8.4	1.9 (1.5,2.3)	214	11.4	152	10.4	68	8.0	10	7.2	444
Severe headache	369	46.5	425	594	7852	62	10.4	1.7 (1.3,2.0)	183	9.7	117	8.0	80	9.4	10	7.2	390
Dizziness	341	42.9	453	594	7852	48	8.1	1.7 (1.3,2.1)	133	7.1	83	5.7	76	8.9	11	8.0	303
Cramp	292	36.8	502	419	7852	42	10.0	1.4 (1.1,1.7)	134	7.1	93	6.4	63	7.4	8	5.8	298
Loss of appetite	238	30.0	556	335	7852	23	6.9	1.4 (1.1,1.7)	105	5.6	83	5.7	49	5.7	9	6.5	246
Abdominal distension	227	28.6	567	332	7852	25	7.5	1.5 (1.2,1.8)	123	6.5	98	6.7	65	7.6	11	8.0	297
Severe nausea and vomiting	197	24.8	597	252	7852	15	6.0	1.3 (1.0,1.6)	106	5.6	84	5.7	50	5.8	5	3.6	245
Anaemia	177	22.3	617	177	7852	14	7.9	1	59	3.1	57	3.9	22	2.6	6	4.3	144
Lack of sleep	135	17.0	659	180	7852	19	10.6	1.3 (1.0,1.6)	71	3.8	48	3.3	35	4.1	4	2.9	158
Blurred vision with a headache	107	13.5	687	143	7852	25	17.5	1.3 (1.0,1.6)	47	2.5	33	2.3	39	4.6	8	5.8	127
Fever	105	13.2	689	139	7852	10	7.2	1.3 (1.0,1.6)	64	3.4	52	3.6	30	3.5	4	2.9	150
Backache	85	10.7	709	105	7852	13	12.4	1.2 (0.9,1.5)	34	1.8	27	1.8	34	4.0	8	5.8	103
Dysuria	80	10.1	714	91	7852	5	5.5	1.1 (0.8,1.4)	36	1.9	21	1.4	16	1.9	4	2.9	77
Shortness of breath	37	4.7	757	42	7852	6	14.3	1.1 (0.8,1.4)	19	1.0	7	0.5	16	1.9	3	2.2	45
Stillbirth	26	3.3	768	26	7852	3	11.5	1	8	0.4	10	0.7	2	0.2	0	0.0	20
Oedema of legs	25	3.1	769	63	7852	10	15.9	2.5 (2.1,2.9)	17	0.9	14	1.0	6	0.7	1	0.7	38
Hypertension (> = 140/90mmHg)	21	2.6	773	21	7852	1	4.8	1.8(1.4,2.2)	37	2.0	45	3.1	10	1.2	2	1.4	94
Severe abdominal pain	17	2.1	777	17	7852	3	17.6	1.0 (0.7,1.3)	8	0.4	4	0.3	8	0.9	4	2.9	24
Abortion	14	1.8	780	14	7852	0	0.0	1	6	0.3	2	0.1	1	0.1	0	0.0	9
Varicose vein	12	1.5	782	21	7852	1	4.8	1.8 (1.4,2.2)	8	0.4	5	0.3	4	0.5	1	0.7	18
Vaginal discharge or itching	10	1.3	784	12	7852	5	41.7	1.2 (0.9,1.5)	7	0.4	3	0.2	5	0.6	2	1.4	17
Vaginal bleeding	6	0.8	788	6	7852	1	16.7	1.0 (0.7,1.3)	3	0.2	2	0.1	6	0.7	1	0.7	12
Oedema of face and hands	6	0.8	788	6	7852	1	16.7	1.0 (0.7,1.3)	2	0.1	2	0.1	1	0.1	1	0.7	6
**Total**	**4016**^**1**^	**92.6**^**2**^		**6705**^**3**^		**553**^**4**^	**8.3**	**1.7(1.3,2.1)**^**6**^	**1882**	**43.4**^**7**^	**1464**	**33.7**	**855**	**19.7**	**138**	**3.2**	**4339**

*Number of episodes of illnesses divided by number of women with illness per row;

^1^Total number of women with illness;

^2^Total number of women with illness divided by total number of women in the study;

^3^Total episodes of illnesses;

^4^Total episodes of use of health services;

^5^Total episodes of use of health services (553) divided by total episodes of illnesses (6705);

^6^Total episodes of illnesses divided by total number of women with illness;

^7^Total number of reasons in each column divided by overall number of reasons (e.g., 1882/4339 for waited for self recovery).

Illnesses that we regarded as severe during pregnancy and needed to be examined by health workers (i.e. severe headache, severe vomiting and nausea, hypertension, anaemia, blurred vision with headache, fever, oedema of leg, severe abdominal pain, vaginal discharge or itching, vaginal bleeding, and oedema of face and hand) accounted for 21.3% (1430 of 6705 episodes of illnesses).

### Anaemia

Of 794 pregnant women, 22.3% (95%CI: 20%, 25%) were anaemic, of which 13.9% (110) was mild, 7.9% (63) moderate, and 0.5% (4) severe. However, 93.5% (95%CI: 92%, 95%) (742/794) of pregnant women did not get iron-folic-acid tablet supplementation. Although 177 of 794 pregnant women were anaemic, the uptake of iron-folic-acid tablet supplementation was only 6% (48 of 794 women).

### Hypertension

The incidence of hypertension was 2.6% (95%CI: 1.7%, 4.0%) (21 of 794 pregnant women); however, the rate of use of health services was only 4.8% (1 of 21 women).

### Determinants of illnesses among pregnant women

Table 4 presents the results of Poisson regression and Prentice-Williams-Peterson (PWP) total time survival analysis of illnesses among pregnant women. In the Poisson analysis, gravidity, history of abortion, and walking distance to access the nearest hospital were statistically significant. In the Cox analysis, history of abortion and walking distance to access the nearest hospital were statistically significant.

**Table 4:** Poisson regression and Prentice-Williams-Peterson (PWP) total time survival analysis of illnesses among pregnant women in rural southern Ethiopia, May 2017–July 2018.

Exposure variables	Poisson regression analysis					Prentice-Williams-Peterson (PWP) total time survival analysis					
	Total no. of events for illnesses (n = 6705)	CRR (95.0%CI)	p-value	ARR (95.0%CI)	p-value	Status of total events (illness as Yes/No) (n = 5558 person-time)		CHR (95.0%CI)	p-value	AHR (95.0%CI)	p-value
						Yes	No				
Women’s age in years	6705	**1.02 (1.01, 1.03)**	**0.001**	-	-	5145	413	1.00 (0.99, 1.01)	0.348	-	-
Women’s age at first marriage in years	6705	0.97 (0.92, 1.03)	0.304	-	-	5145	413	0.99 (0.97, 1.01)	0.174	-	-
Women’s age at first birth in years	6705	1.01 (0.96, 1.05)	0.759	-	-	5145	413	0.99 (0.98, 1.02)	0.818	-	-
Birth interval in years											
2+	3526	1.08 (0.96, 1.21)	0.222	-	-	2646	203	1.01 (0.97, 1.05)	0.737	-	-
< 2	3179	1.0		-	-	2499	210	1.0		-	-
Women’s occupation											
Other (daily labourer, farming, etc.)	564	1.07 (0.87, 1.32)	0.511	-	-	371	49	0.95 (0.87, 1.05)	0.292	-	-
Domestic service	6141	1.0		-	-	4774	364	1.0		-	-
Household wealth index											
Rich	3818	0.97 (0.86, 1.10)	0.663	-	-	3010	217	1.02 (0.98, 1.06)	0.381	-	-
Poor	2887	1.0		-	-	2135	196	1.0		-	-
Total household monthly expenditure											
$30+	5175	1.09 (0.95, 1.26)	0.204	-	-	3857	322	0.99 (0.95, 1.03)	0.594	-	-
<$30	1530	1.0		-	-	1288	91	1.0		-	-
Gravidity											
Multigravida	5440	**1.30 (1.13, 1.50)**	**0.000**	**1.42 (1.02,1.96)**	**0.036**	3962	301	1.02 (0.97, 1.07)	0.495	-	-
Primigravida	1265	1.0		1.0		1183	112	1.0		-	-
Parity											
Multipara	5403	**1.27 (1.10, 1.46)**	**0.001**	-	-	3948	301	1.02 (0.97, 1.07)	0.524	-	-
Nullipara	1302	1.0		-	-	1197	112	1.0		-	-
History of abortion											
Yes	565	**1.44 (1.15, 1.81)**	**0.002**	-	-	329	7	**1.06 (1.01, 1.11)**	**0.011**	**1.06 (1.02, 1.11)**	**0.010**
No	6140	1.0		-	-	4816	406	1.0		1.0	
History of stillbirth											
Yes	399	**1.42 (1.15, 1.76)**	**0.001**	**1.30 (1.03, 1.64)**	**0.026**	259	7	1.06 (1.00, 1.12)	0.064	-	-
No	6306	1.0		1.0		4886	406	1.0		-	-
Type of road to the nearest health facility											
Asphalt	1580	**0.83 (0.72, 0.95)**	**0.009**	-	-	1323	133	0.98 (0.93, 1.02)	0.309	-	-
Others	5125	1.0		-	-	3822	280	1.0		-	-
Walking distance to the nearest health post in minutes											
30+	3347	**1.33 (1.18, 1.49)**	**0.000**	-	-	2352	147	1.03 (0.99, 1.07)	0.126	-	-
<30	3358	1.0		-	-	2793	266	1.0		-	-
Walking distance to the nearest health centre in minutes											
40+	2550	**1.18 (1.05, 1.34)**	**0.007**	-	-	1813	91	**1.04 (1.01, 1.08)**	**0.024**	-	-
<40	4155	1.0		-	-	3332	322	1.0		-	-
Walking distance to the nearest hospital in minutes											
60+	4279	**1.43 (1.27, 1.62)**	**0.000**	**1.39 (1.17,1.64)**	**0.000**	3052	161	**1.06 (1.02, 1.11)**	**0.004**	**1.08 (1.03, 1.14)**	**0.003**
<60	2426	1.0		1.0		2093	252	1.0		1.0	

Significant at P-value <0.05, CRR = crude relative risk, ARR = adjusted relative risk, CHR = crude hazard ratio, AHR = adjusted hazard ratio.

The probability of illnesses among the women who have been pregnant two or more times was 42% higher compared to the women who have been pregnant for the first time (ARR = 1.42; 95%CI = 1.02, 1.96). Those pregnant women who had a history of stillbirth were 30% more likely to have an illness compared to those pregnant women who had no history of stillbirth (ARR = 1.30; 95%CI = 1.03, 1.64). Those pregnant women who walked more than 60 minutes to access the nearest hospital were 39% more likely to have an illness compared to those who walked less than that (ARR = 1.39; 95%CI = 1.17, 1.64).

Regarding the hazard ratios for recurrent events (illnesses), during the follow-up period, the total time approach showed that the risk of illness was 6% higher among pregnant women with a history of abortion than those who had no history of abortion; adjusted hazard ratio (AHR = 1.06; 95%CI = 1.02, 1.11). Furthermore, the risk of illness was also 8% higher among pregnant women who walked more than 60 minutes to access the nearest hospital than those who walked less than that (AHR = 1.08; 95%CI = 1.03, 1.14).

### Utilisation of health services

Over an average follow-up of 9.9 weeks, there was a total of 553 episodes of use of health services (minimum = 1, maximum = 3 episodes per pregnant woman). The incidence rate of episodes of the use of health services was 5 per 100 ill pregnant-woman-weeks (95%CI: 4.5, 5.6) (280 failures in multiple-failure-per-subject data over 5558 total analysis time at risk and under observation). Approximately 41 of 794 pregnant women used health services for at least one type of illness during pregnancy.

The overall utilisation of health services for any illness episodes was only 8.3% (95%CI: 7.6%, 8.9%) (553 episodes of use of health services over 6705 episodes of illnesses). For illnesses that we regarded as severe and needed to be examined by health workers (i.e. severe headache, severe vomiting and nausea, hypertension, anaemia, blurred vision with headache, fever, oedema of leg, severe abdominal pain, vaginal discharge or itching, vaginal bleeding, and oedema of face and hand), only 10.3% used health services (147 episodes of the use of health services over 1430 episodes of illnesses). Furthermore, only two of the six pregnant women with vaginal bleeding used health services. The main reasons for not using health services were that the women thought the illness would heal by itself; women thought the illness was not serious, women could not afford to visit the health institutions, or women lacked confidence in the health institutions (Table 3).

Among 3.9% of pregnant women who had oedema (31 of 794 women), only 32.3% used health services (10 of 31 women). Only two of the mothers who had a severe headache and excessive tiredness or visual disturbance with a severe headache were referred for further treatment. The main reasons for not using health services were that the women thought the illness would heal by itself (43.4%; 1882 of 4339 responses), women thought the illness was not serious (33.7%; 1464 of 4339 responses), women could not afford to visit the health institutions (19.7%; 855 of 4339 responses), or women lacked confidence in the health institutions (3.2%; 138 of 4339 responses) (Table 3).

The rate of the use of health services during anaemia was only 7.9% (14 episodes of the use of health services over 177 episodes of illnesses). The main reasons were that some women could not afford to visit the health institutions (2.6%; 22 of 855 responses), and women lacked confidence in the health institutions (4.3%; 6 of 138 responses) (Table 3).

For the analysis of the association between each of the illnesses and the use of health services, illnesses were used as exposure and use of health services as outcome variables. The overall analysis showed that a significant association existed between illness and use of health services; x^2^ = 11.99, df (1), p-value<0.001. However, analysis of each type of illness and use of health services indicated that heartburn, dysuria, and swelling of face and hand had no significant association.

### Determinants of utilisation of health services

Table 5 presents the Poisson regression and Prentice-Williams-Peterson (PWP) total time survival analysis of the use of health services. In the Poisson analysis, none of the exposure variables were statistically significant. However, in the Cox analysis, history of abortion and walking distance to access the nearest hospital were significantly associated.

**Table 5:** Poisson regression and Prentice-Williams-Peterson (PWP) total time survival analysis on the use of health services for illnesses among pregnant women in rural southern Ethiopia, May 2017–July 2018.

Exposure variables	Poisson regression analysis					Prentice-Williams-Peterson (PWP) total time survival analysis					
	Total no. of events for the use of health services (n = 553)	CRR (95.0%CI)	p-value	ARR (95.0%CI)	p-value	Status of total events (use of health services as Yes/No) (n = 5558 person-time)		CHR (95.0%CI)	p-value	AHR (95.0%CI)	p-value
						Yes	No				
Women’s age in years	553	1.05 (0.98, 1.12)	0.152	-	-	280	5278	1.03 (0.97, 1.09)	0.306	-	-
Women’s age at first marriage in years	553	1.27 (0.97, 1.66)	0.082	-	-	280	5278	1.08 (0.83, 1.41)	0.555	-	-
Women’s age at first birth in years	553	**1.29 (1.08, 1.55)**	**0.006**	-	-	280	5278	1.18 (1.00, 1.39)	0.053	-	-
Birth interval in years											
2+	253	0.86 (0.44, 1.65)	0.640	-	-	119	2730	0.70 (0.38, 1.29)	0.256	-	-
< 2	300	1.0		-	-	161	2548	1.0		-	-
Women’s occupation											
Other (daily labourer, farming, etc.)	45	1.05 (0.38, 2.87)	0.928	-	-	28	392	1.36 (0.50, 3.69)	0.547	-	-
Domestic service	508	1.0		-	-	252	4886	1.0		-	-
Household wealth index											
Rich	315	1.05 (0.55, 2.02)	0.884	-	-	161	3066	0.98 (0.53, 1.80)	0.941	-	-
Poor	238	1.0		-	-	119	2212	1.0		-	-
Total household monthly expenditure											
$30+	490	**2.87 (1.13, 7.32)**	**0.027**	-	-	245	3934	2.31 (0.92, 5.82)	0.076	-	-
<$30	63	1.0		-	-	35	1344	1.0		-	-
Gravidity											
Multigravida	493	1.81 (0.81, 4.07)	0.151	-	-	231	4032	1.43 (0.64, 3.18)	0.378	-	-
Primigravida	60	1.0		-	-	49	1246	1.0		-	-
Parity											
Multipara	485	1.36 (0.61, 3.01)	0.452	-	-	224	4025	1.23 (0.58, 2.63)	0.589	-	-
Nullipara	68	1.0		-	-	56	1253	1.0		-	-
History of abortion											
Yes	95	2.20 (0.85, 5.66)	0.103	-	-	35	301	2.22 (0.91, 5.41)	0.079	**2.50 (1.00, 6.01)**	**0.050**
No	458	1.0		-	-	245	4977	1.0		1.0	
History of stillbirth											
Yes	27	0.92 (0.13, 6.55)	0.934	-	-	7	259	0.51 (0.07, 3.62)	0.501	-	-
No	526	1.0		-	-	273	5019	1.0		-	-
Type of road to the nearest health facility											
Asphalt	198	**2.22 (1.14, 4.31)**	**0.018**	-	-	105	1351	1.69 (0.91, 3.14)	0.097	-	-
Others	355	1.0		-	-	175	3927	1.0		-	-
Walking distance to the nearest health post in minutes											
30+	300	1.06 (0.56, 2.00)	0.867	-	-	147	2352	1.35 (0.74, 2.47)	0.326	-	-
<30	253	1.0		-	-	133	2926	1.0		-	-
Walking distance to the nearest health center in minutes											
40+	205	0.78 (0.39, 1.53)	0.461	-	-	92	1812	0.94 (0.50, 1.78)	0.848	-	-
<40	348	1.0		-	-	188	3466	1.0		-	-
Walking distance to the nearest hospital in minutes											
60+	399	1.34 (0.66, 2.74)	0.417	-	-	196	3017	1.70 (0.88, 3.30)	0.114	**1.91 (1.00, 3.63)**	**0.048**
<60	154	1.0		-	-	84	2261	1.0		**1.0**	

Significant at P-value <0.05, CRR = crude relative risk, ARR = adjusted relative risk, CHR = crude hazard ratio, AHR = adjusted hazard ratio.

Regarding the hazard ratios for recurrent events (use of health services), during the follow-up period, the total time approach showed that the use of health services was three times higher among pregnant women with a history of abortion than those who had no history of abortion; adjusted hazard ratio (AHR = 2.5; 95%CI = 1.00, 6.01). Furthermore, the use of health services was also 91% higher among pregnant women who walked more than 60 minutes to access the nearest hospital than those who walked less than that (AHR = 1.91; 95%CI = 1.00, 3.63).

## Discussion

This study demonstrates that over 90% of rural Ethiopian pregnant women experienced at least one symptom or illness during pregnancy. However, few of them received appropriate care. The Risk factors for both illnesses and utilisation of health services included history of prior pregnancy loss, and whether the pregnant mother lived far away from the health institution.

Being pregnant two or more times in life time was also a risk factors illness among pregnant women. Many of these illnesses were regarded as minor by the pregnant women; however, for potentially severe conditions, such as hypertension, anaemia and vaginal bleeding, the rate of health service utilization was low.

In this study, some of the Poisson regression estimates during illness analysis are different from the Cox-type survival model. Poisson regression estimates had also wider confidence interval than the Cox-type survival model. In addition, Poisson regression analysis of utilisation of health services was not statistically significant. Therefore, interpretation of the results was based on the Prentice, Williams, and Peterson (PWP) total time model as this model is a robust option for recurrent events, and caution should be used when using Poisson regression to analyse recurrent data [29]. The potential reasons for these results are discussed below.

In our study, the rate of illnesses among pregnant women was higher than that found in previously conducted studies in Ethiopia [7], India [32], and Pakistan [33]. This discrepancy could be explained by the fact that our study constituted a cohort study with multiple visits to pregnant women's homes, and the other studies were cross-sectional. However, the incidence of illnesses was similar to 90.3% from population-based studies in Sri Lanka [34]. The Sri Lankan study showed that illnesses considered as minor were judged not to be minor for women. However, as shown by our study, the overall rate of the use of health services for illnesses during pregnancy was low. The reasons for not seeking care during illnesses included a lack of money, a longer time to travel to a health facility, and women lived far from health facilities.

A severe form of illness during pregnancy could include bleeding, anaemia, hypertension, and fever [35]. Pregnant women with these symptoms did not receive the necessary care at the health posts and were not referred for treatment. Indeed, our study determined that only 37 out of 582 pregnant women with bleeding, anaemia, hypertension, and fever used health care services. However, a study from Egypt found that all pregnant women with such illnesses used health care services more often [36]. This difference could be ascribed to the socioeconomic status of the women, a lack of trust in the health institutions, financial constraints, and living far away from the health institution. These findings emphasize the critical needs to identify and address barriers to health service utilization, assess the quality of care, re-train health extension workers about illness identification, and take requisite public health measures.

The burden of anaemia in our study was similar to that of other studies in Ethiopia [37,38], and it was most likely resultant from iron deficiency [39]. Our findings revealed that the uptake of iron-folic-acid tablet supplementation and health service utilization for anaemia were low. This low health service utilization during anaemia may put the women at risk, as they were in a severe condition, as documented in our findings. Possible explanations for this could be that poor pregnant woman may prefer to remain at home due to a longer time to travel to health institutions live far away from health institutions are unable to pay for health services and transportation, and have a lack of trust in health institutions.

In this study, pregnant women with the prior history of pregnancy loss [40] and pregnant women who have had many previous pregnancies [41] had an increased incidence of maternal illnesses. Although the incidence of hypertension was high, few hypertensive pregnant women used health services, as has also been reported from Bangladesh [42]. This finding indicated that pregnant women with the prior history of pregnancy loss, pregnant women who have had many previous pregnancies, and pregnant women who had hypertension could be at higher risk of complications of the pregnant women, her child and low utilisation of health services. In addition, only two of the pregnant women with the illness were referred for further treatment. This could be attributed to that pregnant woman with these illnesses were supposed to incur a cost in order to obtain health care services. Although health care services for pregnant women are free, if pregnant women experience any illness, they have to pay for health care and transportation. Therefore, the Ministry of Health should explore how they could cover these costs.

A non-severe form of illness during pregnancy could include dizziness, abdominal cramp, headache, dysuria, shortness of breath, abdominal distension, lack of sleep, pain in the pelvic area and tiredness, and could affect the day-to-day life of pregnant women [34]. In this study, many pregnant women experienced pain in the pelvic area and tiredness, and they did not use health services. This finding was comparable to a study in rural Bangladesh, which reported that although 87% of pregnant women had illnesses during their pregnancy, 73% of them did not seek any care [9]. This finding was also similar to that reported in an Australian longitudinal study, in which many pregnant women had non-severe illnesses, but 68% did not use health services [43], and in Sri Lanka, 90% did not use health services [34]. The use of health services is not primarily determined by a pregnant woman’s recognition of a problem. A possible explanation is that pregnant women with illnesses were reluctant to seek, and delayed in seeking, health care as pregnant women with symptoms perceived their illness to be minor and may heal by itself. Further delay in seeking care may result from a lack of trust in the health facilities, as well as underlying household poverty which may preclude the use of health services.

A study in Pakistan suggests that older pregnant women are more likely to have illnesses during the pregnancy period [9], which was consistent with our study. The findings from our study revealed that pregnant women daily labourers and farmers are particularly at risk for illnesses during pregnancy. This finding is similar to that reported in a study in which heavy workloads during pregnancy, including long workdays, are associated with illnesses [44]. Those households with higher annual household expenditures are also at risk of catastrophic payments during illnesses [45]. This is also in accordance with our findings. Therefore, in order to alleviate the problem of the failure to utilize needed health care services, the socioeconomic and demographic environment of pregnant women requires additional focus.

The World Health Organization designed a template for how to estimate the incidence of illness during pregnancy based on a cross-sectional study design [2]. However, our results indicate that many illness episodes during pregnancy were not regarded as severe by patients. Consequently, additional work on how to improve the questionnaires may be needed, and one possibility is to include questions about the use of health services.

Our methods of registration of symptoms, based on a recording of associated disability during pregnancy, revealed a high incidence of illness during pregnancy; however, there was low health care service utilization. A community-based illness survey could be employed for early detection, to improve the health of mothers, and to take necessary public measures. In this regard, this study coincides with the World Health Organisation’s matrix for assessing illnesses during pregnancy [2]. The need to measure and count severe or non-severe disabilities reported by pregnant women could assist to address the health needs of pregnant women, and serve as an indicator of access to health care, quality of the health care system, and the possibility of a survey of illnesses to enable more informed decisions.

To the best of our knowledge, this study is the first of its kind in Ethiopia to investigate factors associated with diseases or illnesses during pregnancy and the utilization of health services. It constitutes an antenatal care-based cohort study with repeated measures to identify the determinants of illnesses and subsequent use of health services during pregnancy.

In this study, only those pregnant women who attended antenatal care were recruited, which might constitute a potential selection bias, as a random selection of pregnant women from the communities was not employed. In addition, those pregnant women with less than two antenatal visits were not recruited, and this may have caused a selection bias. However, the proportion of observed (number of pregnant women included in our study) to expected (total number of pregnant women) antenatal care visits in the three kebeles was in agreement with birth registry studies in southwest Ethiopia in which the coverage of maternal health services was approximately 75% [46]. Thus, our study may be representative of pregnant women attending two or more antenatal care visits, but may not be fully representative of pregnant women who had less than two ANC visits or did not attend such services at all. Pregnant women not attending antenatal care could have a higher incidence of illnesses during pregnancy and a lower use of health services. In addition, the recruitment of women was based on ANC attendance and not based on gestational age. As there was a displacement of residents in the study area and its surroundings [47], the number of pregnant women in the analysis was 88.4% due to incomplete data.

## Conclusions

The burden of illnesses during pregnancy is high in rural Ethiopia. Unfortunately, very few ill pregnant women used health care services. Although the Ethiopia Federal Ministry of Health has implemented a health extension programme which has enabled the country to achieve significant improvements in community-based maternal health and healthcare-seeking [48], the findings from this study indicate that much remains to be done, at least in the kebeles. This study provides useful insights concerning illnesses during pregnancy and the use of health services to assist the Ministry of Health to ensure maternal health and strengthen health extension programmes for community-based maternal services to provide a reasonable level of healthcare.

A poor understanding of what severe and non-severe symptoms remain important reasons for the low use of health services. Our study could serve as a basis for more detailed interventions in Ethiopia. Therefore, we recommend that the efforts of the Ministry of Health be directed towards amenable risk factors for immediate clinical and policy interventions on system improvement to increase access and home-based antenatal care services. These intermediate interventions include the promotion of health education in healthcare-seeking that encourages women to seek appropriate and timely care. In addition, long-term interventions could focus on changing the negative effects of poverty.

As we did not study the health system in the context of illnesses during pregnancy, the content and quality of services provided by health extension workers at the community-level remain unassessed. As the health extension program was developed in a context in which maternal health outcomes and coverage of essential health services were very poor [48], examining the knowledge and skills of health extension workers in identifying and managing illnesses during pregnancy could constitute a productive research area in the future.

## Supporting information

S1 Table A table for symptoms of pregnancy-related illnesses and case definition. (DOCX)

S1 Questionnaire.rar Pregnancy-related and utilisation of health services questionnaires in Gedeo language. (RAR)

S2 Questionnaire.rar Pregnancy-related and utilisation of health services questionnaires in Amharic language. (RAR)

S3 Questionnaire.rar Pregnancy-related and utilisation of health services questionnaires in English language. (RAR)
