# Supplementary material for: Utilisation of health services fails to meet the needs of pregnancy-related illnesses in rural southern Ethiopia: A prospective cohort study
Source: PLoS One. 2019 Dec 4;14(12):e0215195. doi: 10.1371/journal.pone.0215195 (PMC6892537; doi:10.1371/journal.pone.0215195)
Supplement: S1 Table — (DOCX) [file pone.0215195.s001.docx]

| **Type** | **Symptoms** | **Signs** | **Definition** |
| --- | --- | --- | --- |
| Spontaneous abortion | Severe abdominal or back pain | Heavy bleeding soaking two or more pads an hour for two hours | A pregnancy is ended before 28 weeks of gestation |
| High fever | Headache | Sweating, chills and shivering | Temperature > 37.5 °C on any day |
| Foul vaginal discharge/itching | Pain, itching, or burning around the vagina | The fluid that flows out the vaginal opening | A fluid with burning, rash, and/or odour due to infection of the genital tract |
| Dysuria | Feeling of pain, or burning upon urination | Cloudy urine | Painful urination |
| A severe headache | Pain on one side or both sides of your head | Sensitivity to light, sounds, | A symptom of pain anywhere in the head |
| Visual disturbances/blurred vision | Hazy, blurred, or double vision. Seeing floating "spider webs" | Swollen, itching, burning, or discharge red eyes | A vision disorder with an impairment of the sense of vision |
| Severe abdominal pain | Discomfort in the upper left or right; middle; or lower left or right abdomen | Swelling and tenderness when you touch the abdomen | Stomach ache |
| A backache | An ache or pain anywhere in the back Pain that worsens with bending, lifting, standing or walking | Inflammation or swelling on the back. Observe back pain, where lying down or resting | Pain in any region of the back |
| Vaginal bleeding | Contractions, abdominal pressure, cramping, and ache in the lower back | Spotting or discharge of blood from vagina | Any bleeding through the vagina during pregnancy |
| Pain in the pelvic area | Pain in the region of women's pelvic area, or groin area | Painful or difficult urination | Pain in the area of the pelvis |
| Nausea and vomiting | Feel nauseous, and abdominal pain | Gagging, retching, choking, involuntary stomach reflexes, the mouth filling with saliva, and need to move or bend over | The sensation of unease and discomfort in the upper stomach with an involuntary urge to vomit |
| Heartburn/regurgitation | Burning sensation or feeling in the chest just behind the breastbone that happens after eating and lasts a few minutes to several hours | Shortness of breath, radiation to the arms or neck, dizziness or a cold sweat. Chest pain, especially after bending over, lying down or eating | Burning sensation in the central chest or upper central abdomen |
| Cramp | Pulling sensation on one or both sides of the abdomen, feel a sharp, stabbing pain, or a dull ache in the lower abdomen. Discomfort in the space between the chest and pelvis, pain may be felt in the abdomen | Notice pain during sneezing or change potions. Tenderness of stomach | Short-lived pains in your lower abdomen |
| Lack of sleep | Excessive daytime sleepiness | Forgetfulness, yawning, moodiness, depressed mood, fatigue, inability to concentrate | The condition of not having enough sleep |
| Fatigue/Tiredness | Headache, dizziness, aching muscles | Sleepiness, slowed reflexes and responses, impaired decision-making and judgment | A subjective feeling of tiredness |
| Loss of appetite | Decreased appetite, reduced the desire to eat | Not wanting to eat,  not feeling hungry, The idea of eating food may make feel nauseous or vomit | The decreased sensation of appetite |
| Varicose vein | Painful, achy, or "heavy feeling" in legs. Burning, throbbing, muscle cramping and swelling in lower legs. Worsened pain after sitting or standing for a long time | Large veins that you can see just under the surface of the skin. Veins are dark purple or blue in colour. Skin discolouration around a vein. Veins appear twisted and bulging; they are often like cords on legs | Veins of the leg that have become enlarged and twisted. Varicose veins are twisted, enlarged veins. |
| Oedema/Swelling of face and hands | Feel uncomfortable. It can even restrict the range of motion in face and wrists. | Puffiness of face and hands, Skin that retains a dimple (pits) after being pressed for several seconds. Stretched or shiny skin. | Swollen face and hands |
| Swelling of legs | Feel uncomfortable. It can even restrict the range of motion in your ankles. | Puffiness of your legs, Skin retains a dimple (pits) after being pressed for several seconds. Stretched or shiny skin | Swollen leg, feet, and ankles |
| Dizziness | Giddiness or a "dizzy spell. Light-headedness,  feeling faint or passing out, spinning, whirling, or motion - either of themselves or of the surroundings, weakness, tiredness, confusion, headache or head pressure or tightness, nausea, or vomiting. | Feeling off balance | Impairment in spatial perception and stability |
| Abdominal distension | The sensation of fullness, abdominal pressure and possibly nausea, pain or cramping | Belching or flatulence, distended or swelling  abdomen | Outward expansion beyond the normal girth of the stomach and waist |
| Dyspnoea/Shortness of breath | Difficulty catching a breath. Chest pain | Noisy, fast, shallow, wheezing breathing. pale and slightly blue skin, especially around the mouth | Feeling like one cannot breathe well enough |
| Anaemia | Easy fatigue and loss of energy. Shortness of breath and headache, Difficulty in concentrating.  Dizziness | Unusually rapid heart beat, pale skin. | A condition that develops when your blood lacks enough healthy red blood cells or haemoglobin |
| Hypertension | Severe headache. Fatigue or confusion. Vision problems. Pounding in chest, neck, or ears. | Difficulty breathing. Irregular heartbeat. | Either a systolic or a diastolic blood pressure measurement consistently higher than an accepted normal value |
